# Not just a picture of a changing treatment landscape: what registry data from Germany add to our knowledge about thermal ablation for kidney tumors

**DOI:** 10.1007/s00330-025-11415-5

**Published:** 2025-02-28

**Authors:** Timo Alexander Auer, Thomas Kröncke

**Affiliations:** 1 Department of Radiology, Charité—Universitätsmedizin Berlin, Augustenburger Platz 1, 13353 Berlin, Germany; 2 Department of Diagnostic and Interventional Radiology, University Hospital Augsburg, Augsburg, Germany

Interest in minimally invasive tumor ablation is at an all-time high, possibly driven by recent studies on thermal ablation (TA) for colorectal liver metastases, which have demonstrated that thermal ablation is comparable to minimally invasive surgery in effectiveness [1]. In fact, TA for renal cell carcinoma (RCC) is also steadily gaining acceptance in various national and international guidelines, driven by numerous recent studies demonstrating oncologic outcomes comparable to nephron-sparing surgery. This has resulted in a rise in the number of thermal ablations performed for RCC in recent years [2–5]. Despite the growing number of procedures and the comparable outcomes to surgery for small renal masses (SRM) up to 4 cm, TA remains classified as an alternative therapy, ranking behind partial nephrectomy in most guidelines [3–7]. In addition to political factors, this is attributed to the quality of data on TA, which primarily comes from single-center and retrospective cohort studies. Registries contribute to our knowledge by offering complementary information to data collected from randomized or observational studies, which may guide further research. There is still no clear consensus on which ablative technique may be superior. A comparison between the traditional hyperthermic methods, radiofrequency ablation (RFA), microwave ablation (MWA), and cryoablation (CA) as a hypothermic technique, is of particular interest in this context.

In this issue of *European Radiology*, Schaarschmidt et al presented real-world data from a large cohort registry study on the current use of percutaneous ablation in renal tumors. Based on a cohort of 1102 patients, the authors examined the technical success rate and complications associated with different thermal ablation techniques [8]. The data collective is one of the largest in Europe offering insight into the clinical practice in Germany and on the treatment results across different ablative techniques. The voluntary nature of the registry as part of a system of quality control introduced by the German Society of Interventional Radiology (DeGIR) is not without limitations: data quality and granularity are variable, and follow-up data is scarce. The authors focused on the strengths of the registry, which lies in the procedural data entered by 92 sites in Germany, and assessed the technical success and complication rates associated with TA for renal tumors measuring 3–4 cm [8]. In line with the current literature, the authors concluded that, in this exploratory registry, the analysis showed that percutaneous TA of SRM is technically feasible with low complication rates. Although the conclusion does not surprise, it becomes intriguing when the authors differentiate the outcomes of individual ablation techniques, distinguishing between heat-based TA and (CA). The most used treatment was RFA (43.5%), followed by MWA (41.9%) and CA (13.3%). Subsequently, when evaluating the technical success and complication rates, it is important to recognize that most cases (86.7%) were treated with heat-based TA, while only a minor portion (13.3%) were treated with CA. Heat-based TA was significantly more technically successful in lesions ≤ 3 cm compared to lesions sized between 3–4 cm, while for CA, no significant difference was reported regarding lesion size. The same pattern is observed in the distribution of complication rates, where complication rates for heat-based techniques increase with tumor size, while no such variation is seen with (CA). Accordingly, the authors concluded that heat-based TAs seem to have lower success rates and higher complication rates in larger tumors, and (CA) potentially be a safe alternative for 3–4 cm sized tumors [8].

In fact, this statement contrasts with much of the existing literature, which often differentiates only between tumors up to 4 cm (T1a) and those between 4 and 7 cm (T1b), while no significant difference was identified between heat-based TA and CA for tumors up to 4 cm. This applies to technical success, complications, and oncological outcomes alike. There are certainly reasons why (CA) might be safer than heat-based TA procedures for larger lesions, primarily due to improved intraprocedural visualization. As a classical multiprobe technique, it also allows for the creation of larger ablation zones. While the increased risk of bleeding during (CA) has been documented and is currently under discussion, our own experience with the new-generation CA systems and their needles does not support this concern. The growing body of data supporting (CA) for T1b tumors, with some individual studies even reporting success for tumors up to 10 cm (T2), reinforces this observation [9]. However, there are no studies demonstrating that cryoablation offers a significant advantage for tumors larger than 4 cm. The authors’ identification of a significant difference in technical outcome for the heat-based at a cutoff of 3 cm to 4 cm is surprising and should be regarded as a new and highly interesting signal. A systematic review on the use of RFA found that tumor size >  3 cm and central tumor location are the major risk factors for treatment failure, while Lam et al also concluded that size alone, without the need for complex scoring systems, may serve as a predictor of incomplete ablation following RFA [10, 11]. The decision to distinguish between tumors of ≤ 3 cm and 3–4 cm is noteworthy and seems to be well-considered, especially considering the international guideline comparisons where this differentiation is not made [3–5]. What all guidelines share is the lack of differentiation between individual TA techniques. While for lesions up to 3 cm, both heat-based TA and CA seem to be equal, the efficacy for tumors 3 cm or larger, as well as for T1b tumors, should at least be taken into consideration.

Overall, it is hoped that ablative procedures will play a more significant role in future guidelines and be considered a treatment of choice alongside surgical options for tumors up to 4 cm. It will be interesting to observe how external beam radiation techniques evolve and what results innovations like histotripsy will yield [12]. We congratulate Schaarschmidt et al for their registry study and the valuable new insights it has provided.
